# Animal Chemistry

**Published:** 1837-10

**Authors:** 


### ANIMAL CHEMISTRY.

### *A Process for discovering Pus in the Blood.* By M. Maudl.

M. M. observes, that ammonia, considered by a great number of physiologists as a proper reagent for the detection of pus, is not so; for, if it changes this liquid into a transparent jelly, it transforms the blood almost in the same manner. The means proposed by M. Maudl consists in separating the fibrine of the blood by heating it with a glass rod. If the blood is pure, and not yet formed into a clot, the rod is soon found covered with an elastic membrane, leaving, when pressed under the finger, a sensation analogous to that which wet elastic gum produces. This membrane, of a red colour, passes, with washing, through a series of tints, less and less deep, and becomes yellowish. If, on the contrary, pus in a very small quantity is found mixed with the blood, it does not form around the rod an entire membrane, but an accumulation of filamentous shreds deprived of elasticity. If the quantity of pus is still more considerable, it neither forms membranes nor filamentous shreds, and the blood left at rest does not form a clot.

*Revue Médicale. Mars,* 1837.

